# An Announcement by the Ministry of Health

**Published:** 1920-05-08

**Authors:** 


### MEDICAL REMEDIES.

### An Announcement by the Ministry of Health.

SOME misunderstanding has arisen as to the objects and scope of the Committee recently appointed by Dr. Addison to consider measures for the effective control of certain therapeutic substances. The terms of reference do not include and have no relation whatever with the subject of patent or proprietary medicines (as those terms are popularly understood), nor is the work of the new Committee in any sense a reduplication of the work of the Select Committee on Patent Medicines appointed in 1912.

"THERAPEUTIC SUBSTANCES."

The "therapeutic substances" now under consideration are recognised remedial substances, the purity and standard of efficacy of which cannot be adequately ascertained by the employment of ordinary chemical tests as can that of the great majority of medicinal substances. This determination can only be done in the substances under consideration in properly equipped laboratories by the employment of biological or physiological methods. The substances in question may be divided into two great classes:—

(1) Bodies such as serums and vaccines, of which a well-known example is the antitoxin given in cases of diphtheria.

(2) Mineral and vegetable bodies of another kind, the most familiar examples of which are salvarsan, the well-known remedy for syphilis, and digitalis, a remedy in certain forms of heart disease.

UNIFORM SYSTEM OF REMEDIES.

At present there is not in this country as there is in some others, any effective supervision and control of the manufacture or standardisation of many of the important agents mentioned above. The work of the Committee is, among other things, to devise measures to co-ordinate and control the various tests and standards employed by firms and persons engaged in the preparation and sale of these substances and to devise a uniform system of standardisation and control the better to guarantee that these agents are what they purport to be and are of an accepted standard of efficacy.

In a few cases, however, *e.g.*, salvarsan, a body of the nature defined above may be the subject of a patent. It is only in such exceptional products, which are usually of foreign origin, that the question of patent medicines will arise at all, and even these bodies have nothing whatever in common with the ordinary run of patent and proprietary medicines with which the public is familiar.

